# Subjective factors affecting prognosis of 469 patients with esophageal squamous cell carcinoma: a retrospective cohort study of endoscopic screening

**DOI:** 10.1186/s12876-022-02399-3

**Published:** 2022-06-28

**Authors:** Jun Nakamura, Noriaki Manabe, Tomoki Yamatsuji, Yoshinori Fujiwara, Takahisa Murao, Maki Ayaki, Minoru Fujita, Akiko Shiotani, Tomio Ueno, Yasumasa Monobe, Takashi Akiyama, Ken Haruma, Yoshio Naomoto, Jiro Hata

**Affiliations:** 1 Division of Endoscopy and Ultrasonography, Department of Clinical Pathology and Laboratory Medicine, Kawasaki Medical School, 2-6-1 Nakasange, Kita-ku, Okayama, 700-8505 Japan; 2 Department of General Surgery, Kawasaki Medical School, Okayama, Japan; 3 Department of Digestive Surgery, Kawasaki Medical School, Kurashiki, Japan; 4 Division of Gastroenterology, Department of Internal Medicine, Kawasaki Medical School, Kurashiki, Japan; 5 Department of Pathology, Kawasaki Medical School, Okayama, Japan; 6 Department of Pathology, Kawasaki Medical School, Kurashiki, Japan; 7 Division of Gastroenterology, Department of Internal Medicine 2, Kawasaki Medical School, Okayama, Japan; 8 Division of Endoscopy and Ultrasonography, Department of Clinical Pathology and Laboratory Medicine, Kawasaki Medical School, Kurashiki, Japan

**Keywords:** Esophageal squamous cell carcinoma, Cancer screening, Prognosis, Symptoms

## Abstract

**Background:**

To date, no in-depth studies have focused on the impact of various clinical characteristics of esophageal squamous cell carcinoma (ESCC), including its association with subjective symptoms, on patient prognosis. We aimed to investigate the clinical factors that affect the prognosis of patients with ESCC and to clarify how subjective symptoms are related to prognosis.

**Methods:**

We retrospectively evaluated the clinical records of 503 consecutive patients with ESCC from April 2011 to December 2019. Six established prognostic factors for ESCC (body mass index, alcohol drinking, cigarette smoking, sex, clinical stage, and age) and subjective symptoms were used to subgroup patients and analyze survival differences. Next, the patients were divided into two groups: a symptomatic group and an asymptomatic group. In the symptomatic group, differences in the incidence of subjective symptoms according to tumor size, tumor location, macroscopic tumor type, and clinical stage were examined. Finally, subjective symptoms were divided into swallowing-related symptoms and other symptoms, and their prognosis was compared.

**Results:**

Multivariate Cox regression analysis identified sex [hazard ratio (HR) 1.778; 95% CI 1.004–3.149; *p* = 0.049], TNM classification (HR 6.591; 95% CI 3.438–12.63; *p* < 0.001), and subjective symptoms (HR 1.986; 95% CI 1.037–3.803; *p* = 0.0386) as independent risk factors for overall survival. In the symptomatic group, the mean time from symptom onset to diagnosis was 2.4 ± 4.3 months. The incidence of subjective symptoms differed by clinical stage, and the prognosis of patients with swallowing-related symptoms was significantly worse than that of patients with other symptoms.

**Conclusion:**

The results of this study suggest that screening by upper gastrointestinal endoscopy, independent of subjective symptoms (especially swallowing-related symptoms), may play an important role in the early detection and improvement of prognosis of ESCC, although further validation in a large prospective study is needed.

## Background

Esophageal cancer is the seventh most common cancer and the sixth leading cause of cancer-related death worldwide. In 2020, an estimated 604,100 new cases of esophageal cancer and 544,076 deaths were reported [1]. In 2019 in Japan, esophageal cancer was the eighth most common cancer among men and the seventh leading cause of cancer-related death, with a mortality rate of 9.4 deaths per 100,000 people [2]. In Europe and the United States, the incidence of esophageal adenocarcinoma is increasing and that of esophageal squamous cell carcinoma (ESCC) is decreasing. In Japan, however, the prevalence of ESCC remains high, and approximately 90% of esophageal cancers are ESCC [3].

In Japan, ESCC is usually detected by upper gastrointestinal (GI) endoscopy. ESCC is an extremely malignant tumor that predominantly affects the mid to lower esophagus, and as it progresses, it causes esophageal obstruction. In the early stages of the disease, esophageal symptoms such as dysphagia may be mild. As the cancer progresses and the esophageal lumen narrows, severe dysphagia may occur. However, a detailed evaluation of the relationship between subjective symptoms and tumor progression as well as prognosis has not been performed in ESCC until now.

Although many studies have shown that age, male sex, body mass index, smoking, alcohol intake, foods containing N-nitroso compounds, hot drinks, red meat intake, and human papillomavirus are the main risk factors for ESCC [4, 5], most patients are diagnosed at an advanced stage. Limited therapies are available for these patients, and their prognosis is very poor. The 5-year overall survival rate is less than 40%, and many patients die within 1 year of diagnosis despite advances in therapy [6]. However, because the prognosis of early-stage ESCC has been shown to be very favorable [7], its detection at an early stage is considered to be of great clinical importance.

In Japan, there is currently no mass screening program for ESCC. This may be due to the fact that the number of patients with ESCC is relatively small compared with the number of patients with gastric, colorectal, lung, breast, and cervical cancer, for which there are organized screening programs. There is also the possibility that endoscopic screening does not improve the prognosis of ESCC because of its extremely rapid progression. The doubling time is often used as a guide to determine the rate of tumor growth. Our previous study showed that the doubling time of gastric cancer was 10.1 months, whereas that of ESCC was 3.3 months, indicating an extremely high tumor growth rate [8].

As described above, ESCC is potentially curable if detected early. With the exception of a study from China in 2020 [9], no report to date has addressed the importance of endoscopic screening for ESCC. The first aim of this study was to investigate the clinical factors that affect the prognosis of patients with ESCC, and the second aim was to elucidate in detail how the subjective symptoms are related to their prognosis.

## Methods

### Patients

In this retrospective study, we evaluated the clinical records of 503 consecutive patients with esophageal cancer who underwent a thorough examination, including definitive diagnosis by pathologists and staging based on the current American Joint Committee on Cancer TNM system [10], at Kawasaki Medical School General Medical Center and Kawasaki Medical School Hospital from April 2011 to December 2019. The first lesion detected during the study period was included in the analysis. If multiple lesions were simultaneously detected, the largest lesion was included. Patients who had previously been treated for ESCC at other hospitals and/or for whom the details of the diagnostic procedure were unknown were excluded. The following data were retrospectively reviewed: clinical manifestations, age, sex, body mass index, history of alcohol consumption, smoking history, reason for endoscopic examination, underlying disease, tumor site, tumor size, histopathological grading, macroscopic tumor type, primary treatment, current American Joint Committee on Cancer TNM classification [10], vital status of patients (survival, death, or loss to follow-up), and duration of follow-up. Six established prognostic factors for ESCC (body mass index, alcohol drinking, cigarette smoking, sex, clinical stage, and age) [11] and subjective symptoms were used to subgroup patients and analyze survival differences. As shown in Table 1, the 5-year survival rate of stage ≥ III ESCC and stage < III ESCC patients differs greatly. Therefore, we divided the clinical stage into two groups: stage ≥ III ESCC and stage < III ESCC.

**Table 1:** Patients’ clinicopathological features

	Our cases (n = 469)
Age	68.8 ± 9.4
Sex (men/women)	385/84 (4.6/1)
Clinical stage of disease (0/I/II/III/IV)	81/76/68/118/126
Treatment* (ESD or EMR/OPE/CT and or RT/BSC)	90/257/150/42
Outcome (alive/dead/unknown)	160/212/97
Observation period (months)	29.8
Drinking (%)	392 (83.6%)
Smoking (%)	376 (80.2%)
DM/HT/DL	80/211/86
Diagnosis opportunity (medical checkup/outpatient consultation due to symptoms)	129/270
History of cancer of other organs (%)	154 (32.8%)
Lesion location (Ce/Ut/Mt/Lt/Ae)	25/91/271/69/10
Macroscopic tumor type (0/1/2/3/4/5)	207/18/124/89/8/23
Histopathological grading (well/moderate/poor/unknown)	91/243/71/64
Cases of stenosis (%)	75 (16.0%)
5-year survival rate	
Stage 0	0.98 (95% CI 0.86–0.99)
Stage I	0.92 (95% CI 0.77–0.98)
Stage II	0.69 (95% CI 0.49–0.83)
Stage III	0.35 (95% CI 0.24–0.47)
Stage IV	0.16 (95% CI 0.07–0.29)

Data are presented as mean ± standard deviation, n, or n (%) unless otherwise indicated

*ESD* endoscopic submucosal dissection, *EMR* endoscopic mucosal resection, *OPE* operation, *CT* computed tomography, *RT* radiation therapy, *BSC* best supportive care, *DM* diabetes mellitus, *HT* hypertension, *DL* dyslipidemia, *Ut* upper thoracic esophagus, *Mt* middle thoracic esophagus, *Lt* lower thoracic esophagus, *M* male, *F* female, *CI* confidence interval

*There was overlap of some treatments

Next, the patients were divided into two groups: a symptomatic group and an asymptomatic group. Each clinical stage group was further divided. The symptomatic group comprised patients who visited one of our hospitals for any upper GI symptom, such as dysphagia, chest pain, or chest discomfort, and were found to have ESCC during upper GI endoscopic examinations, whereas the asymptomatic group comprised patients who underwent upper GI endoscopy for screening purposes and were incidentally found to have ESCC. First, the proportions of the symptomatic and asymptomatic groups among all eligible patients were investigated, and differences in clinical characteristics, pathological findings, and prognosis between the two groups were compared. Next, in the symptomatic group, differences in the incidence of subjective symptoms according to tumor size, tumor location, macroscopic tumor type, and clinical stage were examined. Finally, the patients in the symptomatic group were further divided into two groups: patients with swallowing-related symptoms and patients with other symptoms. Swallowing-related symptoms were defined as symptoms of either dysphagia or discomfort during swallowing. The prognosis was compared between the two groups.

This study was approved by the ethics committee of our hospital (Institutional Review Board no. 3806-1) and was conducted in accordance with the ethical principles related to the Declaration of Helsinki. Neither the patients nor the general public were involved in the design, conduct, reporting, or dissemination of the study plan.

### Statistical analysis

Continuous and categorical variables are presented as mean ± standard deviation and number (%), respectively. Continuous data were compared using the Mann–Whitney U test. Pearson’s χ^2^ test or Fisher’s exact test was used to analyze categorical data and compare proportions. The survival rates of patients were plotted using Kaplan–Meier curves, and differences were evaluated using the log rank test. Cox regression analysis was used to estimate the hazard ratio (HR) and calculate the 95% confidence interval (CI). All statistical analyses were performed using SPSS Version 17.0 (SPSS Inc., Chicago, IL, USA). A *p*-value of < 0.05 (two-sided) denoted a statistically significant difference.

## Results

### Patients’ background

**Fig. 1:**
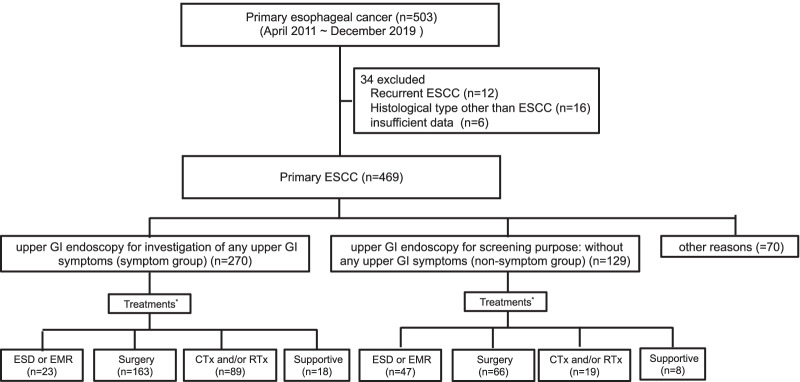
Study flowchart. *ESCC* esophageal squamous cell carcinoma, *GI* gastrointestinal, *ES* endoscopic screening, *ESD* endoscopic submucosal dissection, *EMR* endoscopic mucosal resection, *CTx* chemotherapy, *RTx* radiotherapy. *There was overlap in some treatments

In total, 503 consecutive patients were enrolled from April 2011 to December 2019. Of these, 12 patients who had previously been treated for ESCC, 16 patients with histological types other than squamous cell carcinoma, and 6 patients with insufficient data were excluded. Thus, 469 patients were finally registered in the study. The most common reason for upper GI endoscopy was investigation of any upper GI symptom in 270 (57.6%) patients (symptomatic group), followed by screening in 129 (27.5%) patients (asymptomatic group) and other reasons in 70 (14.9%) (Fig. 1).

The backgrounds of the 469 patients with ESCC are shown in Table 1. The patients’ mean age was 68.8 ± 9.4 years, and there was a male predominance with a male:female ratio of 4.6:1.0. Approximately four-fifths of patients were current or ex-smokers and reported a habitual use of alcohol. Among all patients, 19.4% of ESCCs were located in the upper thoracic esophagus, 57.8% were located in the middle thoracic esophagus, and 14.7% were located in the lower thoracic esophagus. The number of patients with simultaneous multiple lesions in this study group was 72 (15.4%). The breakdown of clinical stages was stage 0 in 81 cases, stage I in 76 cases, stage II in 68 cases, stage III in 118 cases, and stage IV in 126 cases. The 5-year survival rates of patients with each stage were 98%, 92%, 69%, 35%, and 16%, respectively. In total, 52.0% of patients had stage III or IV ESCC at the time of diagnosis. Best supportive care was the treatment of choice for 5.5% of patients. During the mean observation period of 29.8 months, 160 patients survived and 212 died, of whom 144 (67.9%) died of ESCC.

### Clinical factors affecting the prognosis of ESCC

**Fig. 2:**
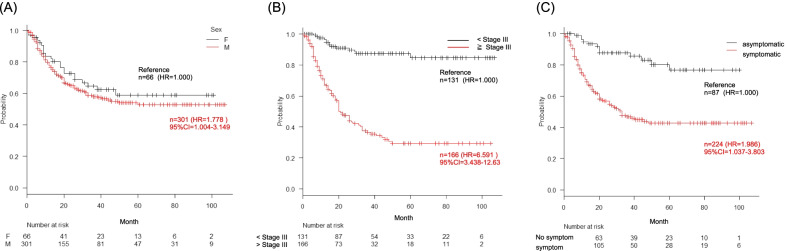
Kaplan–Meier estimates of overall survival according to clinical factors. Illustrated are Kaplan–Meier survival curves, hazard ratios (HRs) including 95% confidence intervals (CIs), patients at risk, and *p*-values of the three independent factors related to the prognostic outcomes in patients with esophageal squamous cell carcinoma. **A** Sex. **B** Stage. **C** Symptoms

Prognostic factors that may affect long-term survival were evaluated and are summarized in Fig. 2 and Table 2. The univariate analysis suggested that male sex, stage ≥ III ESCC, and subjective symptoms tended to be associated with shorter survival. The multivariate Cox proportional hazards regression analysis (Table 2) showed that the independent factors associated with a poor prognosis were male sex (HR 1.778; 95% CI 1.004–3.149; *p* = 0.049), stage ≥ III disease (HR 6.591; 95% CI 3.438–12.63; *p* < 0.001), and subjective symptoms (HR 1.986; 95% CI 1.037–3.803; *p* = 0.0386).

**Table 2:** Possible independent prognostic factors and relative risk

Prognostic factor	Patient, no	Cox regression *p* value	HR	95% CI
Body mass index		0.45		
< 25 kg/m^2^	314			
> 25 kg/m^2^ (reference)	44			
Alcohol drinking		0.556		
Yes	314			
No (reference)	52			
Cigarette smoking		0.865		
Yes				
No (reference)				
Sex		0.048		
Men	301		1.778	1.004–3.149
Women (reference)	66		1.000	
Stage		< 0.001		
< stage III (reference)	131		1.000	
≧ stage III	166		6.591	3.438–12.63
Symptom		0.039		
Yes	224		1.986	1.037–3.803
No (reference)	87		1.000	
Age		0.107		
≧ 75 years old	77			
< 75 years old (reference)	290			

*CI* confidence interval, *HR* hazard ratio (the HR of the reference is defined as 1)

### Comparison between symptomatic and asymptomatic groups

Results of the comparison of the patients’ background factors and clinicopathological findings between the symptomatic and asymptomatic groups are shown in Table 3. There were no significant differences in the patients’ background factors, including sex, age, smoking rate, and drinking rate, between the two groups. The detailed reasons for endoscopic examination in the asymptomatic group were screening in 98 patients, follow-up of GI diseases such as chronic atrophic gastritis in 22, and other purposes in 9.

**Table 3:** Differences in patients’ clinicopathological features between the symptomatic and asymptomatic groups

	Asymptomatic group (n = 129)	Symptomatic group (n = 270)	*P* value
Age	67.9 ± 8.99	68.8 ± 9.19	0.368
Sex (men) (%)	107 (82.9%)	221 (81.9%)	0.889
Alcohol drinking (%)	113 (87.6%)	222 (83.5%)	0.3
Cigarette smoking (%)	101 (78.3%)	217 (81.6%)	0.489
DM (%)	21 (16.3%)	40 (14.8%)	0.766
HT (%)	64 (49.6%)	107 (39.6%)	0.083
DL (%)	24 (18.6%)	44 (16.4%)	0.572
History of cancer of other organs (%)	41 (32.0%)	75 (27.8%)	0.409
Stage of disease (0/I/II /III/IV)	36/30/39/14/10	25/25/39/87/94	< 0.001
Treatment (ESD or EMR/OPE/CT and or RT/BSC)	48/64/10/7	21/157/74/18	< 0.001
Lesion location (Ut/Mt/Lt) (%)	23/54/23	17/61/22	0.381
Macroscopic tumor type (0/1/2/3/4)	98/7/16/7/1	62/10/108/83/7	
Histopathological grading (well/moderate/poor) (%)	22/73/5	17/61/22	0.143
Tumor size (mm)	29.8 ± 20.8	33.8 ± 33.8	< 0.001
Cases of stenosis (%)	1 (4.8%)	61 (38.1%)	< 0.001
Observation period (months)	63.7	34.6	< 0.001

Data are presented as mean ± standard deviation, n, or n (%)

*DM* diabetes mellitus, *HT* hypertension, *DL* dyslipidemia, *ESD* endoscopic submucosal dissection, *EMR* endoscopic mucosal resection, *OPE* operation, *CT* computed tomography, *RT* radiation therapy, *BSC* best supportive care, *Ut* upper thoracic esophagus, *Mt* middle thoracic esophagus, *Lt* lower thoracic esophagus

In the comparison of pathological findings, the lesions were significantly larger and the percentages of patients with stage III and IV cancer were significantly higher in the symptomatic group than in the asymptomatic group; thus, the percentage of patients with ESCC who were available for endoscopic treatment was significantly lower in the symptomatic group than in the asymptomatic group (7.8% vs 37.2%, respectively; *p* < 0.001). No significant differences were identified in tumor location, macroscopic tumor type, or histopathological grading.

With respect to differences in the clinical prognosis between the symptomatic group and asymptomatic group, the Kaplan–Meier survival curve is shown in Fig. 2C. The 5-year survival rate was significantly higher in the asymptomatic group than in the symptomatic group [asymptomatic group: 74% (95% CI 59%–84%) vs symptomatic group: 47% (95% CI 40%–54%), *p* < 0.01].

### Relationship between swallowing-related symptoms and prognosis of ESCC

**Fig. 3:**
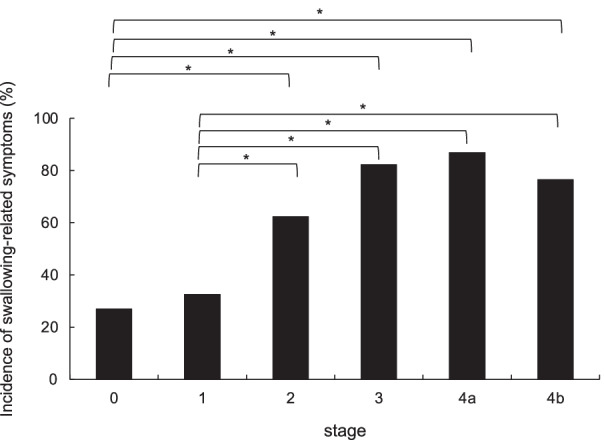
Percentage of patients with swallowing-related symptoms according to clinical stage of esophageal squamous cell carcinoma. *p < 0.01. The frequency of swallowing-related symptoms ranged from 26.8 to 32.5% in patients with stage 0 and/or stage I disease with a high probability of cure and was significantly lower than in patients with stage ≥ III disease

Subjective symptoms were observed in 270 (57.6%) patients. The symptoms were, in order of frequency, swallowing-related symptoms in 186 (68.9%) patients, chest pain in 26 (9.6%), chest discomfort in 14 (5.2%), appetite loss in 8 (3.0%), and body weight loss in 7 (2.6%). The highest frequency of swallowing-related symptoms was 26.8%–32.5% in patients with stage 0 and/or stage I cancer with a high possibility of receiving curative treatment, which was significantly lower than the frequency of symptoms in patients with stage ≥ III cancer (Fig. 3). Patients with esophageal stricture on upper GI endoscopy had significantly more swallowing-related symptoms than those without esophageal stricture (91.7% with stricture vs 51.4% without stricture, *p* < 0.001). Furthermore, when the tumors were classified by macroscopic tumor type, swallowing-related symptoms were identified in 31.6% of the patients with type 0 tumors but were significantly more common in patients with other tumor types (64.7%–90.9%, *p* < 0.001).

**Fig. 4:**
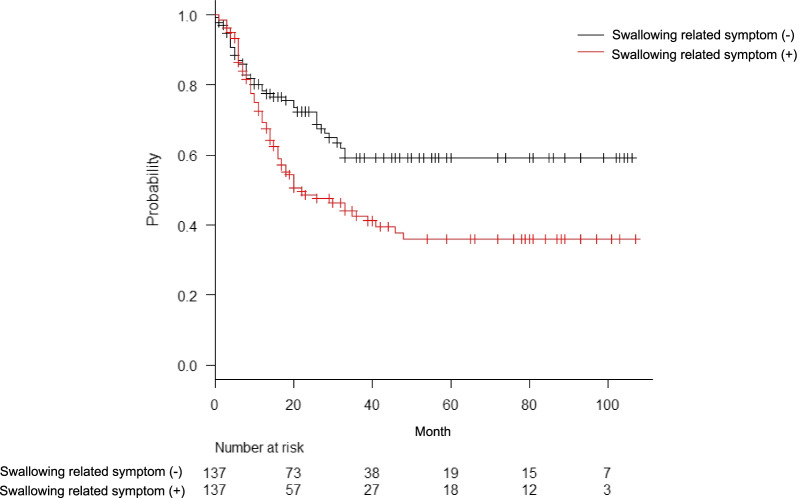
Kaplan–Meier estimates of overall survival in patients with and without swallowing-related symptoms. The prognosis of patients with swallowing-related symptoms was significantly worse than that of patients without such symptoms (*p* = 0.034)

When we investigated the differences in prognosis between patients with swallowing-related symptoms (defined as dysphagia and discomfort during swallowing) and patients with other symptoms, we found that the 5-year survival rate of patients with swallowing-related symptoms was significantly worse, as shown in the Kaplan–Meier survival curve in Fig. 4 [swallowing-related symptoms group: 36% (95% CI 26%–46%) vs other symptoms group: 59% (95% CI 48%–68%), *p* < 0.01]. Furthermore, the mean time from symptom onset to diagnosis was 2.4 ± 4.3 months.

## Discussion

This study showed three clinically important results. First, male sex, TNM classification of ≥ 3, and subjective symptoms were independent risk factors for overall survival of ESCC in Japan. Second, the mean time from symptom appearance to diagnosis was short (2.4 ± 4.3 months), and most patients were difficult to treat endoscopically at the time of symptom appearance. Third, the prognosis of patients with swallowing-related symptoms was significantly worse than that of patients with other symptoms.

In our study, TNM classification of ≥ 3 was identified as a prognostic factor. Mariette et al. identified three favorable prognostic factors: low pT stage, pN0 stage, and surgery during the study period [12]. In a multivariate analysis by Akgul et al. progression of pathological stage had a negative effect on survival [13]. These reports support our results. However, in the present study, there was a sex difference in prognosis. A recent study by Luo et al. indicated that sex was an independent prognostic factor in Chinese patients with ESCC who underwent definitive radiotherapy, with better survival outcomes in women than men [14]. In our study, 108 (23.0%) patients received radiotherapy. Previous studies have shown that androgen exposure can facilitate the proliferation of human ESCC cells and that activation of androgen receptors may promote progression of ESCC [15, 16]. The relationship between androgen levels and prognosis in ESCC patients will be determined in future prospective studies. However, Zhang et al. assessed the role of sex in the prognosis of ESCC in Chinese and in Caucasian patients in the United States and found that it was not an independent prognostic factor in these patients [17], although they did not provide specific information about variables and treatment modalities, which might have affected the analysis. Therefore, the prognostic significance of sex in ESCC should be interpreted cautiously at present, and a further large prospective study will be necessary to clarify this point.

In this study, subjective symptoms, especially dysphagia, were identified as a prognostic factor. A recent study by Zang et al. in residents aged 40–69 years of a region with a high incidence of upper GI cancer showed that subjects with high livestock/poultry meat intake, low fruit and vegetable consumption, and typical symptoms were at higher risk of ESCC, and they speculated on an association between tooth loss and oral hygiene and esophageal disease [18]. We did not examine tooth loss or oral hygiene in our present study, and these points will be investigated in the future. Although it has been empirically shown that many patients with ESCC have already progressed by the time subjective symptoms appear, there have been no reports detailing the relationship between subjective symptoms and their prognosis. In this study, we reconfirmed for the first time that endoscopic screening according to patients’ subjective symptoms is not effective. The survival rate of patients diagnosed after the onset of symptoms has reportedly remained unchanged during the past few decades despite advances in treatment methods [6], which is consistent with our study results. The risk of ESCC is high in men of advanced age, and even higher in heavy drinkers, people who develop flushing of the face after drinking, and smokers. Patients with head and neck cancer, achalasia, and corrosive esophagitis are also considered to be at high risk [4, 19]. Patients with ESCC in this study had also these risk factors. Therefore, endoscopic screening of high-risk groups, without excessive reliance on subjective symptoms, may be important for the early detection of ESCC, which can be treated effectively and with minimal invasion [20].

Unfortunately, most cases of ESCC are detected at an advanced stage of the disease; the 5-year survival rate is less than 20% in developed countries and less than 5% in many developing countries, where most cases of ESCC occur [7]. In this study, 52.0% of consecutive patients with ESCC had advanced cancer, and their 5-year survival rate was 0.211 (95% CI 0.155–0.274), which is considered very poor. However, research has shown that patients with early-stage cancer, which can be treated endoscopically or surgically, have a much better 5-year survival rate of 80%–90% than those with advanced stage [21]. When the patients with ESCC in this study were limited to those with stages 0 or I cancer, the 5-year survival rate was 0.92–0.98, which is consistent with previous reports. Therefore, the main goal of ESCC screening is to detect ESCC at an early stage, allowing for curative treatment. Several studies have demonstrated that endoscopic screening programs in high-risk populations are associated with early detection, reduced mortality, and improved cost-effectiveness. A community assignment study with 10 years of follow-up in an endemic area of China showed a 33% reduction in ESCC-related cumulative mortality in intervention communities that offered one Lugol’s screening session to adults aged 40–69 years [22]. Similar results were observed in another large retrospective study of a high-risk Chinese population [23]. In response to these findings, screening programs have been proposed for high-risk populations worldwide [24]. Screening programs are being further promoted for the treatment of esophageal squamous dysplasia and early ESCC. Unfortunately, because of the low incidence of ESCC, the effectiveness of population-based cancer screening for ESCC has not been demonstrated in Japan; therefore, no organized screening for ESCC is being conducted at present. We believe that our finding that the prognosis of patients varies greatly depending on the reason for upper GI endoscopy in 469 patients with ESCC in Japan is clinically significant. The results of this study may provide evidence to promote efficient endoscopic screening of Japanese patients with ESCC in the future.

This study had several limitations. First, it was a retrospective cohort study conducted at two institutions. Therefore, it was not possible to investigate individual physicians’ experiences or access the upper GI endoscopy findings in individual residential areas. Prospective studies must be designed to address this issue. Second, the possibility of referral filter bias cannot be ruled out. Such bias would increase the number of patients in the asymptomatic group. However, the characteristics of the patients in the present study were not significantly different from those of the patients in the national survey of the Japan Esophageal Society [25], and we believe that our data represent the current status of ESCC without bias. Third, among all patients, 38 (8.1%) had risk-free ESCC, i.e., women with no history of alcohol consumption or smoking. The number of patients with subjective symptoms at the time of consultation was 29 (76.3%), which was significantly higher than that of patients with ESCC with any risk. We believe that screening of these patients will also be a clinical issue to be considered in the future. Fourth, in the present study, in patients with multiple lesions, only the largest tumor was included in the analysis. This could be considered a factor that might affect the results of the study. The number of patients with simultaneous multiple lesions in this study group was 72 (15.4%). This figure was consistent with a previous large study in which a proportion of patients had simultaneous multiple lesions in ESCC [26], confirming that there was no bias in our study population. A previous study by Pesko et al. showed that the tumor stage in patients with multiple tumors was determined by the main tumor stage and was not affected by the associated lesions [27]. Thus, our inclusion of only the largest tumor of patients with multiple lesions in the analysis is reasonable.

## Conclusions

Taken together, the results of this study suggest that screening by upper GI endoscopy, which does not rely on subjective symptoms (especially swallowing-related symptoms), may play an important role in the early detection and prognosis improvement of ESCC. Nevertheless, validation in large-scale prospective studies is needed.

## Data Availability

The datasets used and/or analyzed during the current study are available from the corresponding author (NM) on reasonable request. Neither the patients nor the general public were involved in the design, conduct, reporting, or dissemination of the study plan. For informed consent, opt-out was adopted.

## Abbreviations

CI: Confidence interval
ESCC: Esophageal squamous cell carcinoma
GI: Gastrointestinal
HR: Hazard ratio
